# Development and Examination of the Psychometric Properties of the Social Perception of Artificial Intelligence in Healthcare Scale in the Turkish Context: Evidence From Hatay Province

**DOI:** 10.3389/ijph.2026.1609194

**Published:** 2026-02-25

**Authors:** Fatma Nuray Kuşcu Şahin

**Affiliations:** Hatay Health Services Vocational School, Hatay Mustafa Kemal University, Antakya, Türkiye

**Keywords:** artificial intelligence, attitudes, healthcare delivery, psychometric measurement, scale development

## Abstract

**Objectives:**

The rapid spread of artificial intelligence (AI) in healthcare has increased interest in how the public views and trusts these technologies. However, tools designed to measure these perceptions in the Turkish context remain limited. This study aimed to develop a valid and reliable scale to assess public perceptions of AI in healthcare.

**Methods:**

An initial set of 41 items was created based on the literature and expert input. Data were collected from 404 adults in Turkey and divided into two groups. Exploratory factor analysis was conducted in the first group, followed by confirmatory factor analysis in the second group to test the factor structure, validity, and reliability of the scale.

**Results:**

Exploratory factor analysis showed that the scale has a three-factor structure reflecting attitudes and acceptance, trust, and perceived usefulness. This structure explained 74.35% of the total variance. Confirmatory factor analysis supported this model with an acceptable level of fit, and the results also showed that the scale had strong internal consistency.

**Conclusion:**

The SPAIHS is a psychometrically sound instrument for assessing public perceptions of AI in healthcare.

## Introduction

Artificial intelligence (AI) has recently played a decisive role in the digitalisation of healthcare services. AI is used in many areas of healthcare, ranging from medical imaging and clinical decision-support systems to the analysis of large health datasets and population health management. With the help of deep learning, AI can now interpret medical images with an accuracy similar to that of human experts, highlighting its growing role in clinical practice [1].

AI can process health data in digital systems and help predict the likelihood of disease. It can also model different treatment options and their possible outcomes, which improves the overall efficiency of healthcare services [2]. Because of these capabilities, AI is no longer seen as just a technical add-on. It is now viewed as a structural change that shapes how healthcare is delivered, supports clinical decision making, and improves the patient experience [3]. In Turkey, artificial intelligence is increasingly being used in healthcare management, especially in areas such as resource planning, clinical decision support, digital transformation, and public health. At the same time, its growing use has raised concerns about ethics, accountability, transparency, and how the healthcare workforce will adapt [4]. This rapid, AI-driven transformation not only improves the efficiency of healthcare services but also draws attention to how the public perceives and makes sense of these changes [5].

Studies show that people’s views on AI in healthcare are shaped not only by how well it performs in medical decision making but also by concerns about the reduced role of human involvement [6]. Research also suggests that people feel more uneasy when medical decisions are made by machines, and they are often reluctant to accept systems that might replace human judgment, especially in critical or life-saving situations [7]. These findings suggest that even though AI is widely seen as useful and capable, many people are still hesitant to place full trust in it. This hesitation is shaped by ethical concerns, worries about data privacy, and a range of psychological and emotional factors.

When people think about AI and their attitudes toward it, trust stands out as one of the most important issues. Uncertainty about how algorithms work, the possibility of errors, unclear responsibility, potential bias, and concerns about data privacy can all weaken people’s trust in these systems [8]. Research suggests that people are more likely to trust AI when its decisions are easier to understand and when the way the system works is more transparent [9].

However, even when AI produces highly accurate technical results, this alone is not enough to earn the public’s full trust. When it comes to their health, people also want to feel understood and listened to, something they usually expect from human interaction [10]. In this context, even though AI is generally seen as beneficial, building full trust in these systems remains difficult at the moment. The idea of trust, which lies at the heart of this discussion, cannot be understood from a single point of view. Instead, it is shaped by several factors, including privacy, transparency, the risk of errors, the desire to stay in control, and perceptions of fairness. For this reason, trust should not be seen as a single, simple concept. It is a multidimensional perception shaped by issues such as privacy, transparency, fairness, a sense of control, and the possibility of errors [5].

Research on attitudes toward AI shows that people often experience both positive feelings, such as hope, and negative feelings, such as anxiety, at the same time. For example, one study found that although many people are willing to benefit from the opportunities AI offers in healthcare, they also have serious concerns about fully adopting it, mainly because of unclear issues related to responsibility, ethics, and data privacy [11]. In a similar way, other studies have shown that people’s views of AI are shaped by their past experiences with healthcare. They are also influenced by how much individuals value privacy and confidentiality, their general acceptance of technology, and their concerns about risk [12, 13]. These findings show that public attitudes toward AI are diverse and complex, and they cannot be explained only in technical terms.

Studies examining patients’ attitudes toward AI suggest that trust or distrust is not determined solely by expectations of technological performance. Trust is also shaped by how safe patients feel during their care, whether they worry about possible bias in algorithms, concerns that AI might increase costs, fears about the protection of personal data, and the sense that their autonomy could be threatened [14].

Supporting this view, one study found that patients showed only limited trust in medical decisions made solely by AI. However, their trust increased noticeably when they were told that a physician had reviewed the AI’s recommendations [15]. These findings suggest that people’s views of AI are shaped not only by how well it performs in clinical settings but also by a wider set of psychological and social factors.

International studies [5, 16] have examined attitudes toward AI, including trust and acceptance, across different population groups. However, research that looks at these issues in a multidimensional way at the societal level is still very limited in the Turkish context. This gap in the literature provided the basis for the present study. Accordingly, the study aimed to develop a comprehensive tool to measure how society perceives artificial intelligence in healthcare.

The aim of this study was to develop a valid and reliable scale to measure how people perceive the use of artificial intelligence in healthcare. In particular, the study focused on people’s attitudes toward AI, how much they trust it, and how useful they believe it to be. The study also examined whether the scale reflects these different aspects of public perception in a clear and consistent way.

## Methods

### Study Design and Participants

This psychometric study was conducted using a cross-sectional research design to examine societal perceptions of artificial intelligence (AI) in healthcare. Data were collected in Hatay, a province located in the Mediterranean Region of Turkey.

Individuals aged 18–60 who were literate, able to speak and understand Turkish, residing in Turkey, and willing to participate voluntarily were included in the study. Based on expert opinion, individuals over the age of 60 were excluded due to potential limitations in technology use.

In scale development studies, it is recommended that the sample size should be at least five to ten times the number of items [17]. For the 41-item draft scale, this corresponded to a target sample size of 205–410 participants. A total of 404 individuals were included in the study, which met this recommended range.

### Data Collection Instruments

#### Personal Information Form

The personal information form consisted of nine items addressing age, gender, marital status, education level, employment status, income evaluation, frequency of digital technology use in daily life, level of knowledge about artificial intelligence, and whether participants had ever used AI-based health technologies such as the e-Nabız personal health record system (Ministry of Health, Turkey), step counters, or smart watches. These questions were designed to provide a detailed overview of participants’ personal and technological profiles in line with the aims of the study.

#### Social Perception of AI in Healthcare Scale (SPAIHS)

The initial item pool of the scale was developed through a comprehensive review of national and international literature on the use of AI in healthcare. Searches conducted in PubMed, Scopus, Web of Science, and Google Scholar using keywords such as “artificial intelligence,” “AI in healthcare,” “public perception,” “trust in AI,” “privacy concerns,” “technology acceptance,” and “perceived usefulness” revealed key themes related to perceived usefulness, trust and privacy, risk and uncertainty, and attitudes and acceptance toward AI.

In constructing the item pool, empirical findings reported in the literature, conceptual explanations, results from review studies, theoretical frameworks in healthcare AI and digital health, and the researcher’s prior academic experience in this field were taken into consideration. These sources were compared to generate original items representing each theme, differentiate overlapping content, and ensure the use of accessible language appropriate for individuals with varying levels of literacy. As a result, a theoretical pool of 41 items was created.

To assess content validity, the draft scale was reviewed by a panel of six experts, including two associate professors specializing in measurement and evaluation, three assistant professors working in healthcare AI and digital health, and one Turkish language specialist.

The draft scale and an evaluation form based on the Davis method [18] sent to the experts, who were given 10 days to complete their review. The experts suggested limiting the age range to 60 years, removing unnecessary conjunctions or words, correcting expression errors, and adapting the language to ensure comprehensibility for the general population. The relevant items were revised in line with these recommendations. The I-CVI and S-CVI/Ave values calculated using the Davis method were within acceptable ranges. The scale was designed in a five-point Likert format, scored as 1 = “Strongly Disagree,” 2 = “Disagree,” 3 = “Neutral,” 4 = “Agree,” and 5 = “Strongly Agree.” Following expert evaluation and content validity analysis, the scale items were subjected to factor analysis to assess construct validity.

### Data Collection

Data were collected online via Google Forms between 1 August and 15 September 2025 using a cross-sectional design. Convenience and snowball sampling methods were employed. During the data collection process, the survey link was shared through the researcher’s social and professional networks, as well as via WhatsApp and social media platforms, and posted in various online community groups. Participants were kindly asked to forward the survey link to other individuals who might be willing to participate. This approach aimed to reach adult individuals residing in Hatay Province, Turkey. Participation was entirely voluntary, and respondents could proceed with the survey only after providing informed consent.

Eligibility criteria included being 18–60 years of age, living in Turkey, and being able to read and understand Turkish. Participation was voluntary, and respondents could proceed with the survey only after selecting the statement, “I voluntarily agree to participate in this study.”

The survey consisted of two sections: a personal information form and the Social Perception of AI in Healthcare Scale (SPAIHS). To protect participants’ privacy, no identifying information such as IP addresses or personal identifiers was collected. The survey link was shared with approximately 650 individuals, and a total of 404 completed responses were obtained. On average, participants took about 8 min to complete the survey.

### Data Analysis

IBM SPSS 26 and AMOS 24 were used for the analyses. The total sample of 404 participants was randomly split into two equal groups of 202. The first group was used for exploratory factor analysis to identify the underlying structure of the scale. Before this analysis, sampling adequacy was checked using the Kaiser–Meyer–Olkin (KMO) measure and Bartlett’s test of sphericity [19, 20].

During the EFA, factor loadings, eigenvalues, explained variance, communalities, and item–factor relationships were examined, and items were revised based on the observed structure. To confirm the factor structure identified through EFA, Confirmatory Factor Analysis (CFA) was conducted on Group 2, an independent subsample. Model fit was evaluated using commonly recommended fit indices (e.g., CMIN/df, IFI, TLI, CFI, RMSEA), and the compatibility of the model with the theoretical structure was assessed.

To support construct validity, Composite Reliability (CR) and Average Variance Extracted (AVE) values were calculated to assess convergent validity. Discriminant validity was examined by comparing the square roots of the AVE values with the correlations between the factors. Internal consistency was evaluated using Cronbach’s alpha for both the overall scale and each subdimension. A significance level of p < 0.05 was used for all item analyses [21].

### Research Ethics

The study was conducted in accordance with the principles of the Declaration of Helsinki. Prior to data collection, ethical approval was obtained from the Scientific Research and Publication Ethics Committee of Mustafa Kemal University, Social and Human Sciences (meeting date: 01.08.2025; meeting number: 10; decision no: 07). All participants provided informed consent by reading the consent statement and selecting the option “I voluntarily agree to participate in this study” before beginning the survey.

## Results

### Socio-Demographic Characteristics of the Participants

A total of 404 participants were included in the study. Regarding age distribution, 49.3% were between 18 and 29 years, 38.9% were between 30 and 44 years, and 11.9% were between 45 and 60 years. Of the participants, 55% were women, 52.2% were single, and 59.9% were university graduates. Additionally, 52% reported being employed, and 60.9% described their income level as moderate.

A total of 74.5% of participants indicated that they used digital technologies continuously in daily life, and 32.2% stated that they had an advanced level of knowledge about artificial intelligence. In contrast, 54.5% reported that they rarely used AI-based health technologies (Supplementary Table S1).

### Construct Validity: Exploratory Factor Analysis (EFA) (N = 202)

To assess the suitability of the data for factor analysis, the Kaiser–Meyer–Olkin (KMO) measure of sampling adequacy and Bartlett’s Test of Sphericity were examined. The KMO value was 0.902, and values above 0.80 are described in the literature as indicating “very good” sampling adequacy. Bartlett’s Test of Sphericity yielded χ^2^(820) = 8376.904, p < 0.001, demonstrating that the correlations among variables were appropriate for factor analysis. Based on these results, the dataset was considered suitable for conducting the EFA [22].

The results of the EFA indicated that the scale consisted of a three-factor structure, and the factor loadings are presented in Table 1. In naming the factors, the shared conceptual meaning of the items grouped under each dimension, as well as relevant theoretical insights from the literature, were taken into account. The first factor consisted of items A35, A36, A40, A39, A32, A37, A38, and A41, and was labeled “Attitudes and Acceptance.” Factor loadings ranged from 0.679 to 0.955, and this factor alone explained 41.41% of the total variance. The second factor comprised items A26, A28, A29, A27, A25, A23, and A30, and was labeled “Trust,” as it reflected participants’ confidence in AI. Factor loadings ranged from 0.665 to 0.905, explaining 24.77% of the total variance. The third factor included items A2, A5, A6, A10, A9, A7, and A11, and was labeled “Perceived Usefulness,” as it represented perceptions of AI’s contribution to healthcare. Factor loadings ranged from −0.556 to −0.957, accounting for 8.16% of the variance. Together, the three factors explained 74.35% of the total variance, demonstrating that the scale supports the theoretically proposed structure [19]. Based on these results, the findings indicate that the factor structure of the scale is consistent with the theoretically proposed model.

**TABLE 1:** Factor loadings and explained variance of the scale (Hatay, Turkey, 2025).

Scale items	CVI	F1	F2	F3
A35	1.00	0.955	​	​
A36	1.00	0.942	​	​
A40	1.00	0.907	​	​
A39	0.83	0.831	​	​
A32	1.00	0.790	​	​
A37	1.00	0.742	​	​
A38	1.00	0.689	​	​
A41	1.00	0.679	​	​
A26	1.00	​	0.905	​
A28	1.00	​	0.902	​
A29	1.00	​	0.896	​
A27	0.83	​	0.859	​
A25	1.00	​	0.838	​
A23	1.00	​	0.802	​
A30	0.83	​	0.665	​
A2	0.83	​	​	−0.560
A5	1.00	​	​	−0.957
A6	1.00	​	​	−0.925
A10	0.83	​	​	−0.718
A9	1.00	​	​	−0.702
A7	1.00	​	​	−0.587
A11	0.83	​	​	−0.556
Eigenvalue	​	9.112	5.450	1.797
% Of variance	​	41.417	24.773	8.167
Cumulative %	​	**41.417**	**66.190**	**74.358**

F1, Attitudes and Acceptance; F2, Trust; F3, Perceived Usefulness; CVI, Content Validity Index. Items with factor loadings above 0.40 were retained. AVE, CR, and Cronbach’s alpha values indicate strong construct validity and reliability. Bold values indicate the cumulative percentage of variance explained by the extracted factors.

During the EFA, a total of 17 items were removed based on factor loadings, cross-loadings, and theoretical consistency. Two items (A14 and A24) were excluded because their factor loadings were below 0.40. Several items (A18, A21, A22, A17, A33, and A34) were removed because they loaded on more than one factor and blurred the factor structure. The remaining items (A1, A3, A4, A8, A13, A15, A16, A19, and A20) were excluded because they did not fit well with the theoretical framework of the scale.

As a result of these steps, the EFA yielded a 24-item, three-factor structure. In the subsequent CFA, two additional items (A12 and A31) were removed due to high error covariances and modification indices that negatively affected model fit. Thus, a final 22-item, three-dimensional scale that was both statistically and theoretically robust was obtained. No reverse-coded items are included in the scale.

### Confirmatory Factor Analysis (CFA) (N = 202)

**FIGURE 1:**
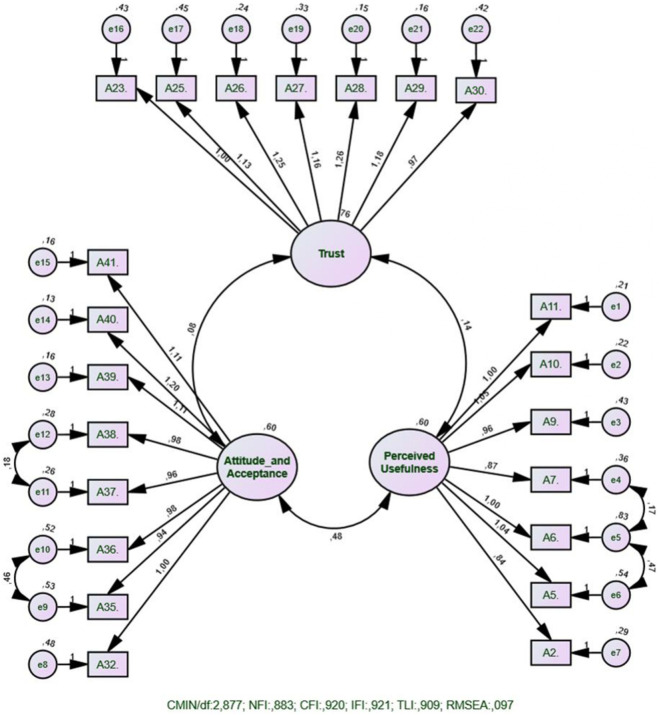
CFA path diagram of the scale (N:202) (Hatay, Turkey, 2025).

The validity of the three-factor structure identified through EFA was tested using Confirmatory Factor Analysis (CFA), as shown in Figure 1. The analysis indicated the following model fit indices: χ^2^/df = 2.877, CFI = 0.920, TLI = 0.909, IFI = 0.921, NFI = 0.883, RMSEA = 0.097, and RMR = 0.066.

Overall, the CFI, TLI, and IFI values were above 0.90, indicating an acceptable fit of the model [23, 24]. The χ^2^/df ratio and RMR value also supported this conclusion. Although the NFI value was slightly below the ideal level, this index is known to be sensitive to sample size and model complexity, so it was not considered a sufficient reason to reject the model [25, 26]. The RMSEA value was within the range considered acceptable in the literature [27].

### Reliability

The internal consistency reliability of the scale was evaluated using Cronbach’s alpha coefficients. According to commonly used guidelines, Cronbach’s alpha values above 0.70 indicate acceptable reliability, and values above 0.90 indicate very strong internal consistency [28, 29].

In this study, the Cronbach’s alpha coefficient was 0.952 for the first factor, 0.938 for the second factor, and 0.927 for the third factor. The alpha coefficient for the overall scale was 0.917, indicating that the items consistently measure the same construct at a high level. All alpha values were above 0.90, indicating that the three subdimensions of the scale were internally consistent and reliable.

As shown in Table 2, positive correlations were observed among the three subdimensions of the scale—Attitudes and Acceptance (AA), Trust (TR), and Perceived Usefulness (PU)—in the expected directions. The strong correlation between AA and PU (r = 0.804) suggests that individuals who perceive AI as useful are more likely to adopt it and develop positive attitudes toward its use. In contrast, the low correlations of the Trust subdimension with both AA (r = 0.114) and PU (r = 0.203) indicate that trust operates more independently from the other dimensions.

**TABLE 2:** Convergent and discriminant validity statistics (Hatay, Turkey, 2025).

Subdimension	AA	TR	PU	CR	AVE	CA
AA	0.792	0.114	0.804	0.954	0.668	0.952
TR	0.114	0.870	0.203	0.956	0.758	0.938
PU	0.804	0.203	0.801	0.927	0.609	0.927

AA, Attitudes and Acceptance; TR, Trust; PU, Perceived Usefulness; CR, Composite Reliability; AVE, Average Variance Extracted; CA, Cronbach’s Alpha.

The √AVE values used to assess discriminant validity showed that each subdimension explains its own items better than it explains the items of other subdimensions. For each factor, the √AVE value was higher than its correlations with the other factors, demonstrating that discriminant validity was achieved [22, 30]. Thus, the subdimensions were confirmed to be conceptually distinct from one another.

To evaluate convergent validity, the AVE values (ranging from 0.60 to 0.76) were examined and found to exceed the acceptable threshold of 0.50. This finding indicates that the items within each subdimension adequately represent their respective constructs [31].

The high values observed in both Cronbach’s alpha (CA) and Composite Reliability (CR) analyses (CA: 0.927–0.952; CR: 0.927–0.956) indicate that the scale possesses a highly consistent and reliable structure [20, 32]. The highest reliability values were observed in the “Attitudes and Acceptance” and “Trust” subdimensions, indicating that the items within these dimensions are highly consistent with one another.

Overall, these findings demonstrate that the scale possesses both convergent and discriminant validity and that all three subdimensions support the theoretically proposed structure. Together, these results indicate that the scale shows strong reliability as well as satisfactory convergent and discriminant validity.

As shown in Table 3, participants generally demonstrated positive attitudes toward the use of artificial intelligence in healthcare (X̄ = 3.58; SD = 0.61). Among the subdimensions, the “Attitudes and Acceptance” dimension had the highest mean score (X̄ = 4.20), indicating that participants broadly embraced healthcare-related AI applications and showed a favorable inclination toward their use.

**TABLE 3:** Descriptive statistics of the scale (Hatay, Turkey, 2025).

Scale and subdimensions	Number of items	Mean	SD	Skewness	Kurtosis
SPAIHS (total)	22	3.58	0.61	−0.523	1.306
Attitudes and acceptance	8	4.20	0.84	−1.113	0.619
Trust	7	2.50	0.99	0.661	−0.184
Perceived usefulness	6	3.95	0.79	−0.739	0.131

The “Perceived Usefulness” dimension also had a high mean score (X̄ = 3.95), suggesting that participants view AI as functional, beneficial, and capable of improving healthcare services. In contrast, the mean score for the “Trust” dimension was considerably lower (X̄ = 2.50). This finding implies that although participants hold positive attitudes toward AI-based health applications, they remain cautious regarding issues such as privacy, data security, and confidentiality.

All Skewness and Kurtosis values for the scale and its subdimensions were within the range of −1.5 to +1.5, indicating that the assumption of normal distribution was met. Overall, these findings show that participants perceive AI applications as useful and acceptable, yet exhibit a more cautious stance regarding the trust dimension [33].

## Discussion

This study examined the validity and reliability of the SPAIHS, a scale developed to measure societal perceptions of artificial intelligence in healthcare. Participants generally reported positive views about the use of AI in healthcare. Perceived usefulness and acceptance were particularly high. However, trust scores were lower. This shows that seeing AI as beneficial does not necessarily mean trusting it. This pattern is consistent with what has been described as the medical AI paradox, in which people recognize the benefits of AI, such as speed and accuracy, yet remain reluctant to place full trust in it [7, 34].

The three-factor structure of the SPAIHS aligns with previous scale development studies demonstrating that attitudes toward AI are multidimensional. Similar patterns have been observed in studies measuring nursing students’ attitudes toward AI. These studies have identified dimensions such as benefit, perceived risk, and willingness to use AI [35]. Studies among dentistry students report a similar pattern. Although AI is seen as a tool that can accelerate treatment, concerns remain about professional roles and data privacy [36]. These results show that the dimensions of the SPAIHS are both statistically sound and theoretically well grounded.

The high Perceived Usefulness scores observed in this study are consistent with international evidence suggesting that AI contributes to more accurate diagnoses, facilitates patient monitoring, and enhances the speed of healthcare delivery. A recent meta-analysis showed that AI performs especially well in imaging and early diagnostic applications. This has contributed to a more positive public perception [37]. An analysis of social media content similarly reported that the majority of posts related to medical AI were positive and conveyed a sense of optimism [38]. Several studies also show that the public expects AI to accelerate healthcare processes and reduce errors [5]. These results, in line with the existing literature, indicate that participants believe AI to be a functional and practical tool in healthcare.

In contrast, the low scores observed in the Trust dimension suggest ongoing concerns regarding privacy, the possibility that data may be used for purposes beyond their original intent, and uncertainties about data security. Privacy is not merely a technical matter. It is a multilayered construct that includes cultural, social, and ethical dimensions. Several studies show that trust in healthcare-related AI depends on more than technical performance [39]. Transparency, data protection, accountability, and ethical regulation are also important. Trust in AI systems does not rely only on accuracy. People also want clear explanations about how the system works. They want to know who controls the data and how it is used [40]. Studies also show that responsibility is a major concern [41, 42]. Many people are unsure who would be held accountable if an AI system makes a mistake.

When the Turkish context is considered, the reasons behind the prominence of these concerns become clearer. The health informatics system in Turkey has long operated in a centralized structure. This has led to citizens experiencing frequent and obligatory interactions with digital health platforms such as e-Nabız and MHRS. This structure makes it easier to adopt new technologies. However, it also raises concerns about who controls health data, how it is processed, and how it may be used in the future. Recent legal and ethical analyses have emphasized the need for greater clarity in this area. This includes issues such as the secondary use of personal health data, anonymization procedures, and cross-border data transfer [43, 44]. In addition, the fact that trust in government institutions and trust in digital applications are not at the same level in Turkey contributes to individuals approaching the use of artificial intelligence in healthcare with greater caution.

The results of this study show that perceptions of AI in healthcare in Turkey are similar to those in many other countries. They fall between technological optimism and caution driven by privacy concerns. Participants strongly believe that AI will add value to healthcare services; however, they become more apprehensive when the processing of their own health data is involved. As noted in international research, improving AI solely at the technical level is not sufficient to enhance public trust. For individuals to feel confident in AI systems, data processing practices must be clearly explained. The basis of system decision-making should be understandable. Accountability in the event of an error must be defined, and ethical frameworks should be transparent and publicly known [39]. Clearly defining these frameworks and communicating them to the public are critically important for strengthening societal trust and facilitating the integration of AI into healthcare services.

In this study, data were mainly collected through WhatsApp and social media. This likely increased the participation of people who actively use digital technologies. The literature indicates that people who are more immersed in digital tools tend to adopt AI applications more readily [45, 46]. Therefore, the high scores observed in the “Attitudes and Acceptance” and “Perceived Usefulness” dimensions may be related to the digitally advantaged nature of the sample. Individuals who are more enthusiastic about technology may show lower levels of trust. They tend to be more sensitive to issues such as privacy, data security, and the functioning of algorithms [47, 48]. This pattern may also help explain why the Trust dimension showed lower scores in our study. Additionally, research indicates that studies involving older adults or groups with limited digital experience typically report lower levels of technology acceptance [49, 50]. Therefore, caution should be exercised when generalizing these results to the broader population, and future research should include more diverse samples.

The results obtained in this study should be interpreted in light of certain sampling-related considerations. Although participation was not formally restricted to residents of a single province, the data collection process was conducted through social networks and online communities centered in Hatay Province. As a result, the sample may reflect regional and contextual characteristics specific to Hatay Province. Convenience and snowball sampling were used together with online recruitment. This may have increased the number of participants who are more digitally engaged and familiar with technology. These factors may limit the generalizability of the findings to the broader Turkish population. Therefore, the results should be interpreted with caution.

Future studies are encouraged to validate the scale using more diverse samples.

These samples should be drawn from different regions and sociodemographic backgrounds.

### Strengths and Limitations

One of the main strengths of this study is the systematic development and psychometric evaluation of a scale specifically designed to assess societal perceptions of artificial intelligence in healthcare. The use of a sufficiently large sample and the division of the dataset into independent subsamples for exploratory and confirmatory factor analyses contribute to the robustness of the scale’s factor structure. In addition, the simultaneous reporting of multiple reliability and validity indicators provides strong evidence for the internal consistency and construct validity of the instrument.

Despite these strengths, several limitations should be considered. Data collection was conducted via social networks and online communities largely centered in Hatay Province, which may have introduced a degree of geographic concentration and could limit the generalizability of the findings to the wider Turkish population. Moreover, the reliance on convenience and snowball sampling combined with online recruitment may have resulted in the overrepresentation of individuals with higher levels of digital engagement. Finally, the cross-sectional design precludes the assessment of temporal changes in perceptions of artificial intelligence in healthcare.

### Conclusion

This study comprehensively evaluated the psychometric properties of the SPAIHS, a scale developed to measure societal perceptions of artificial intelligence (AI) in healthcare. The three-factor structure of the scale, which includes attitudes and acceptance, trust, and perceived usefulness, was supported by both statistical analyses and theory. The outcomes of the exploratory and confirmatory factor analyses support the scale’s construct validity, while the high Cronbach’s alpha, CR, and AVE values indicate strong internal consistency and convergent validity.

Participants generally expressed positive attitudes toward the use of AI in healthcare, with particularly high scores in the usefulness and acceptance dimensions. However, the comparatively lower trust scores suggest that technological optimism coexists with caution regarding privacy and data security. This pattern highlights the need for more transparent and explanatory policies concerning the processing and protection of health data in the context of Turkey.

The SPAIHS provides a reliable way to assess how the public views and trusts artificial intelligence in healthcare across different dimensions. The scale has the potential to contribute meaningfully to monitoring public opinion, planning educational and awareness initiatives, informing policy development, and guiding strategies aimed at enhancing the acceptance of AI applications in healthcare. Future studies applying the scale across different cultures, age groups, and socio-demographic profiles will enable the production of comparative data and support a broader understanding of the societal impacts of AI in healthcare.

## Data Availability

The data supporting this study’s findings are available from the corresponding author upon reasonable request.
