# Supplementary material for: Development and Examination of the Psychometric Properties of the Social Perception of Artificial Intelligence in Healthcare Scale in the Turkish Context: Evidence From Hatay Province
Source: Int J Public Health. 2026 Feb 25;71:1609194. doi: 10.3389/ijph.2026.1609194 (PMC12975613; doi:10.3389/ijph.2026.1609194)
Supplement: Supplementary file 1 [file Supplementaryfile1.pdf]

Supplementary Material 1: Sociodemographic characteristics of the participants (Hatay, Turkey. 2025).

Sociodemographic variable	Groups	N	%
Age	18–29 years	199	49.3
	30–44 years	157	38.9
	45–60 years	48	11.9
Gender	Female	231	57.2
	Male	173	42.8
Marital status	Married	193	47.8
	Single	211	52.2
Educational level	Primary Education	13	3.2
	Secondary Education	30	7.4
	High School	78	19.3
	University	242	59.9
	Postgraduate	41	10.1
Employment status	Employed	253	62.6
	Student	75	18.6
	Retired	16	4
	Unemployed	60	14.9
Income level	Low	66	16.3
	Medium	246	60.9
	High	92	22.8
	Nadiren	30	7.4
	Ara sıra	73	18.1
	Sürekli	301	74.5
Level of knowledge about artificial ıntelligence	None	16	4
	Little	58	14.4
	Moderate	103	25.5
	Sufficient	97	24
	Very Good	130	32.2
Use of aı-based health technology	No	42	10.4
	Yes, infrequently	264	65.3
	Yes, regularly	98	24.3
**Total**		**404**	**100**
