# Supplementary material for: Development and Examination of the Psychometric Properties of the Social Perception of Artificial Intelligence in Healthcare Scale in the Turkish Context: Evidence From Hatay Province
Source: Int J Public Health. 2026 Feb 25;71:1609194. doi: 10.3389/ijph.2026.1609194 (PMC12975613; doi:10.3389/ijph.2026.1609194)
Supplement: Supplementary file 2 [file Supplementaryfile2.pdf]

Supplementary Material 2: Items and factor structure of the Social Perception of Artificial Intelligence in Healthcare Scale (Hatay, Turkey. 2025).

	**Social Perception of AI in Healthcare Scale (SPAIHS)**	**Strongly Disagree**	**Disagree**	**Neutral**	**Agree**	**Strongly Agree**
	**Factor 1 – Attitudes and Acceptance**					
1	I can easily understand the use of artificial intelligence.					
2	I find it easy to understand artificial intelligence applications (e.g., e-Nabız).					
3	I find it easy to use artificial intelligence applications (e.g., e-Nabız).					
4	I support the use of artificial intelligence in healthcare.					
5	I believe that the use of artificial intelligence improves healthcare services.					
6	I am willing to use healthcare applications supported by artificial intelligence (e.g., smart wearable devices).					
7	I recommend artificial intelligence–supported healthcare applications to people around me (e.g., pedometer apps).					
8	I find it worthwhile to spend time researching and learning about healthcare applications supported by artificial intelligence.					
	**Factor 2 – Trust**					
**9**	I believe that the privacy of my health data is protected by artificial intelligence.					
**10**	I believe that artificial intelligence keeps my identity information confidential and uses my health data only for the purpose of improving health.					
**11**	I believe that my health information is secure with artificial intelligence.					
**12**	I believe that artificial intelligence is more reliable than humans in protecting my health information.					
**13**	I believe that the use of artificial intelligence protects the privacy of my health data.					
**14**	I believe that my health information will remain confidential when processed by artificial intelligence.					
**15**	I believe that artificial intelligence manages information about my health better than a healthcare professional.					
	**Factor 3– Perceived Usefulness**					
**16**	I believe that artificial intelligence facilitates the work of healthcare professionals.					
**17**	I believe that the use of artificial intelligence in healthcare reduces patients' waiting time.					
**18**	I believe that the use of artificial intelligence accelerates the delivery of healthcare services.					
**19**	I believe that benefiting from artificial intelligence in healthcare represents an important advancement.					
**20**	I believe that artificial intelligence can help prevent diseases.					
**21**	I believe that the use of artificial intelligence leads to accurate clinical decisions (e.g., diagnosis and treatment).					
**22**	I believe that artificial intelligence is a functional and practical tool for addressing health problems.					

SCALE EVALUATION

Attitudes and Acceptance Factor: The Attitudes and Acceptance dimension consists of Items 1–8, and no reverse-coded items are included in this subscale. The Cronbach's alpha coefficient for this dimension was calculated as 0.927. This factor reflects perceptions regarding the ease of understanding and using artificial intelligence applications, the extent to which individuals consider it worthwhile to allocate time to these technologies, and their beliefs about the role of artificial intelligence in improving healthcare services. Higher scores indicate a more positive attitude and a higher level of acceptance, whereas lower scores reflect limited acceptance or reluctance.

Trust Factor: The Trust dimension consists of Items 9–15, and no reverse-coded items are included. The Cronbach's alpha coefficient for this subscale was found to be 0.938. This dimension measures perceptions regarding the protection of personal health data by artificial intelligence, the provision of privacy, and the maintenance of data security. Higher scores indicate that participants perceive artificial intelligence systems as reliable in terms of data security, whereas lower scores reflect skepticism or limited trust regarding the protection of health data by artificial intelligence.

Perceived Usefulness Factor: The Perceived Usefulness dimension consists of Items 16–22, and no reverse-coded items are included in this subscale. The Cronbach's alpha coefficient for the Perceived Usefulness subscale was calculated as 0.952. Higher scores on this dimension indicate that participants believe that artificial intelligence provides significant usefulness in the delivery of healthcare services and contributes positively in terms of early diagnosis, speed, efficiency, and service quality. Lower scores indicate the perception that the usefulness of artificial intelligence in healthcare services is limited.
