# Supplementary material for: Development and Examination of the Psychometric Properties of the Social Perception of Artificial Intelligence in Healthcare Scale in the Turkish Context: Evidence From Hatay Province
Source: Int J Public Health. 2026 Feb 25;71:1609194. doi: 10.3389/ijph.2026.1609194 (PMC12975613; doi:10.3389/ijph.2026.1609194)
Supplement: Supplementary file 3 [file Supplementaryfile4.pdf]

Supplementary Material 4:STROBE Checklist– (Hatay, Turkey. 2025).

STROBE Item	Recommendation	Section in Manuscript
1	Indicate the study design in the title or abstract	Title; Methods – Study Design
2	Provide background and rationale	Introduction
3	State specific objectives	Methods – Aim
4	Present key elements of study design early	Methods – Study Design
5	Describe the setting, locations, and dates	Methods – Data Collection
6	Describe eligibility criteria and selection of participants	Methods – Study Design and Participants
7	Clearly define outcomes and measurements	Methods – Data Collection Instruments
8	Describe data sources and measurement methods	Methods – SPAIHS
9	Describe efforts to address potential bias	Discussion – Strengths and Limitations
10	Explain how study size was determined	Methods – Sample Size
11	Explain handling of quantitative variables	Methods – Data Analysis
12	Describe statistical methods	Methods – Data Analysis
13	Report numbers of participants	Results – Sociodemographic Characteristics
14	Give characteristics of study participants	Results – Sociodemographic Characteristics
15	Report outcome data	Results
16	Give main results	Results
17	Discuss limitations	Discussion – Strengths and Limitations
18	Provide interpretation of results	Discussion
19	Discuss generalisability	Discussion – Generalizability paragraph
20	Give source of funding	Declarations – Funding
21	Describe ethical approval	Ethics Statement
