# Supplementary material for: Development and Examination of the Psychometric Properties of the Social Perception of Artificial Intelligence in Healthcare Scale in the Turkish Context: Evidence From Hatay Province
Source: Int J Public Health. 2026 Feb 25;71:1609194. doi: 10.3389/ijph.2026.1609194 (PMC12975613; doi:10.3389/ijph.2026.1609194)
Supplement: Supplementary file 4 [file Supplementaryfile3.pdf]

Supplementary Material 3: Removed Items and Reasons for Exclusion (Hatay, Turkey. 2025).

Item Code	Stage of Removal	Primary Reason for Exclusion	Explanation
A1	EFA	Minor factor formation	The item formed a separate and theoretically heterogeneous minor factor that was not compatible with the overall structure of the scale.
A3	EFA	Minor factor formation	The item did not conceptually align with the core dimensions of social perception of artificial intelligence.
A4	EFA	Minor factor formation	The item deviated from the theoretical framework and exhibited a fragmented structure.
A8	EFA	Minor factor formation	The item showed content overlap with other items and provided limited unique contribution.
A13	EFA	Minor factor formation	The item did not adequately support the multidimensional structure and weakened conceptual coherence.
A15	EFA	Minor factor formation	The item was not sufficiently related to the intended dimensions of social perception.
A16	EFA	Minor factor formation	The item demonstrated weak theoretical consistency within the scale structure.
A19	EFA	Minor factor formation	The item was overly general and showed low discriminative power.
A20	EFA	Minor factor formation	The item was evaluated as providing limited specific contribution to the measured construct.
A14	EFA	Low factor loading	The item was removed because its factor loading was below the acceptable threshold of 0.40.
A24	EFA	Low factor loading	The item did not adequately represent its associated latent factor.
A18	EFA	Cross-loading	The item loaded at similar levels on multiple factors, compromising factorial clarity.
A21	EFA	Cross-loading	The item weakened the discriminant structure of the factors.
A22	EFA	Cross-loading	The item caused overlap between factors and reduced structural distinctiveness.
A17	EFA	Cross-loading	The item created conceptual ambiguity by representing more than one dimension.
A33	EFA	Cross-loading	The item displayed theoretical heterogeneity across factors.
A34	EFA	Cross-loading	The item was evaluated as disrupting overall factor integrity.
A12	CFA	High error covariance	The item negatively affected model fit due to high error covariance.

A31	CFA	High modification index	The item was removed due to conceptual redundancy and a high modification index that adversely affected model parsimony.
